# Relationship between engagement with the impossible task, cognitive testing, and cognitive questionnaires in a population of aging dogs

**DOI:** 10.3389/fvets.2022.1052193

**Published:** 2023-01-04

**Authors:** Michael Z. Khan, Alejandra Mondino, Katharine Russell, Beth Case, Gilad Fefer, Hope Woods, Natasha J. Olby, Margaret E. Gruen

**Affiliations:** Department of Clinical Sciences, College of Veterinary Medicine, North Carolina State University, Raleigh, NC, United States

**Keywords:** canine cognitive dysfunction syndrome, canine cognition, frustration, aging, dogs

## Abstract

**Introduction:**

The aim of this study was to evaluate the engagement of aging dogs with a cognitively challenging and potentially frustrating task (the impossible task). Based on previous observations, we predicted that dogs showing signs of cognitive impairment in other cognitive tests and owner-completed questionnaires would show reduced engagement with the task.

**Methods:**

In this task, dogs were shown a piece of food in a clear container that they could not open; time spent interacting with the container and the experimenter was measured. While the impossible task has not been used as a measure of frustration, the parameters of the test design creates a potential frustrate state, making this assessment appropriate. Thirty-two dogs enrolled in a longitudinal aging study participated in the study. Owners were asked to complete two cognitive dysfunction screening questionnaires (Canine Dementia Scale [CADES] and Canine Cognitive Dysfunction Rating Scale [CCDR]) as well a questionnaire assessing general frustration. Dogs participated in multiple measures of cognitive function as well the impossible task.

**Results:**

Latency to disengage from the impossible task was faster for dogs with higher total (more impaired) CADES (*p* = 0.02) and total CCDR (*p* = 0.04) scores. Latency to disengage also correlated with decreased performance in cognitive tests observing social cues (*p* = 0.01), working memory (*p* ≤ 0.001), spatial reasoning and reversal learning (*p* = 0.02), and sustained attention (*p* = 0.02).

**Discussion:**

The high correlation with several cognitive measures and the ease of administration of this test makes it a useful tool in evaluating canine cognitive dysfunction syndrome, however it is unclear if increased frustration or other cognitive processes are contributing to the observed changes.

## Introduction

Behavioral changes as dogs (*Canis familiaris*) age can be among the first clinical signs of underlying disease (1). Up to an estimated 68% of dogs experience some behavioral change as they age, with the highest prevalence in the oldest dogs (2, 3). Apart from normal aging changes, dogs can develop pathologic cognitive dysfunction, often referred to as Canine Cognitive Dysfunction Syndrome (CCDS). Dogs with this condition generally present with multiple behavioral changes including disorientation, changes to social interactions, altered sleep/wake cycle, house-soiling, changes in activity, worsening memory, deficiency in learning, and increased anxiety (4–11). Previous research has demonstrated that owners also note a decline in vision, hearing, and olfaction in older dogs (12, 13). Studies in canine cognition have also demonstrated that older dogs tend to perform worse than younger dogs in tasks involving attention, learning, and problem-solving (4, 13–18). Questionnaires have been developed to assess these behavioral changes and attempt to categorize dogs with CCDS based on the severity of their signs (1, 9, 19). While we can subjectively quantify the behavioral changes using these validated owner-completed instruments, it is difficult to assess the underlying etiology behind the behavior changes (1, 19). From a clinical perspective, it is important to understand the motivation behind a behavior in order to develop a complete and thorough treatment plan (20, 21).

As dogs lose sensory and cognitive abilities, we can assume their ability to predictably impact their environment also diminishes. These cognitive and sensory changes can lead to situations where the dog may expect a different outcome or be unable to predict an outcome for a given circumstance; unmet expectations can lead to the negative emotional state known as frustration (22). Frustration-related behaviors have been documented and consist primarily of vocalizations, but also include pacing, sniffing, reorientation, and distancing from the frustrating stimuli (23). These frustration-related behaviors may initially increase in intensity if the expected outcome is not met but will eventually cease when given enough time (22, 24). Frustration has been implicated in several behavioral problems in dogs ranging from redirected behaviors, repetitive behaviors, and aggression toward conspecifics (20, 25). A continued state of frustration may lead to an increase in arousal and an overall negative affective state (26) with physiologic similarities comparable to fear/anxiety and pain (27). The role of frustration in the development of the anxiety-related behavioral changes seen in dogs with CCDS has not been previously studied but may be a motivating factor among these dogs.

This research is hindered by the limited tools available to assess canine frustration. The Canine Frustration Questionnaire (CFQ) has been published to assess overall and subtypes of canine frustration (28). In the development of this owner-completed questionnaire, it was found that dogs whose owners scored as low in frustration coping skills also demonstrated a higher degree of frustration behaviors during tasks where the dogs' expectations were not met (either by restraining the dog or by placing them behind a barrier) (29). However, the tasks used did not always correlate with questionnaire results and are not easy to perform in the clinic; development of an alternative task would be beneficial for assessing frustration in dogs. The impossible task is a cognitive test that closely mirrors previous tests of frustration. In the impossible task, a dog is able to obtain a food reward from an easily manipulated container over a set of initial trials. During the test trial, the container is sealed, and the dog's ability to access the reward during the testing period is removed. This creates a frustrating situation that does not resolve over the 90-s of the trial (30). The impossible task has previously been used to assess differences in canine communication and problem-solving strategies while interacting with their environment (30, 31). Previous research has shown that the main strategies for “solving” the impossible task are interacting with the container, looking to the human experimenter, or a combination of these (30). Studies in aging dogs have shown older dogs will gaze at their owners (acting as experimenters) less than younger dogs during the impossible task (32), however, total time engaging with the task using either strategy has not been studied. The impossible task requires very little equipment and can be performed in a short period of time (~5 min). Further, this task allows the dog to be unrestrained and does not require prior training.

As part of ongoing work on neuroaging, client-owned dogs in our studies participate in a series of cognitive tests that require varying amounts of effort and cognitive flexibility (13). It was observed early in testing that as dogs did not receive an anticipated reward from a cognitive test (due to a delay or an incorrect response), they would start to display signs of frustration (barking, whining, and pacing) and might ultimately cease to participate even when the task would change. The current study aimed to evaluate the engagement with impossible task as a measure of motivation to engage in a difficult and frustrating task. Specifically, we hypothesized that dogs who interact with the impossible task less and disengage with the test more quickly will show reduced cognitive performance in their cognitive testing scores, owner perception of their cognition, and show increased owner perceived frustration.

## Materials and methods

### Study population

Dogs in this study came from a population of client-owned senior dogs currently enrolled in a longitudinal study of neuro-aging at the NC State University College of Veterinary Medicine (CVM) (13). Dogs were selected and given the same clinical assessment and questionnaires as previously described (13). All dogs were systemically healthy, and met the inclusion criteria regarding mobility, vision, and absence of focal neurological deficits. In addition to the age of the dog their calculated expected life span was assessed using the following formula [13.62 + (0.0702^*^ht) – (0.0538^*^wt.)] (33). Fractional lifespan was then calculated by taking their total age and diving by the calculated lifespan. The questionnaires included two assessments of cognitive status using the Canine Dementia Scale (CADES) (19) and the Canine Cognitive Dysfunction Rating (CCDR) (9). These were given within 1 week of participating in the current study. These clinical metrology instrument asks owners to quantify the frequency of common CCDS signs, with a higher overall score indicating more severe cognitive decline. The CADES questionnaire can additionally be assessed by subsection of questions regarding spatial disorientation and confusion, social interactions, house soiling behaviors, and sleep behaviors. In addition to the standard battery of questionnaires, the Canine Frustration Questionnaire (CFQ) (28) was also given to owners. The CFQ asks owners to subjectively rate a dog's frustration through a series of questions based on a Likert scale, with a higher score indicating a higher degree of general frustration.

All owners were given the details of the study methods and signed an informed consent before the dog participated in any experiment. All cognitive tests were performed in the designated cognitive testing room at North Carolina State University. The rooms were vacuumed or mopped before testing each dog and water was available free choice for each dog during their testing sessions. All procedures were approved by the North Carolina State University Institutional Animal Care and Use Committee.

### Impossible task

**Figure 1:**
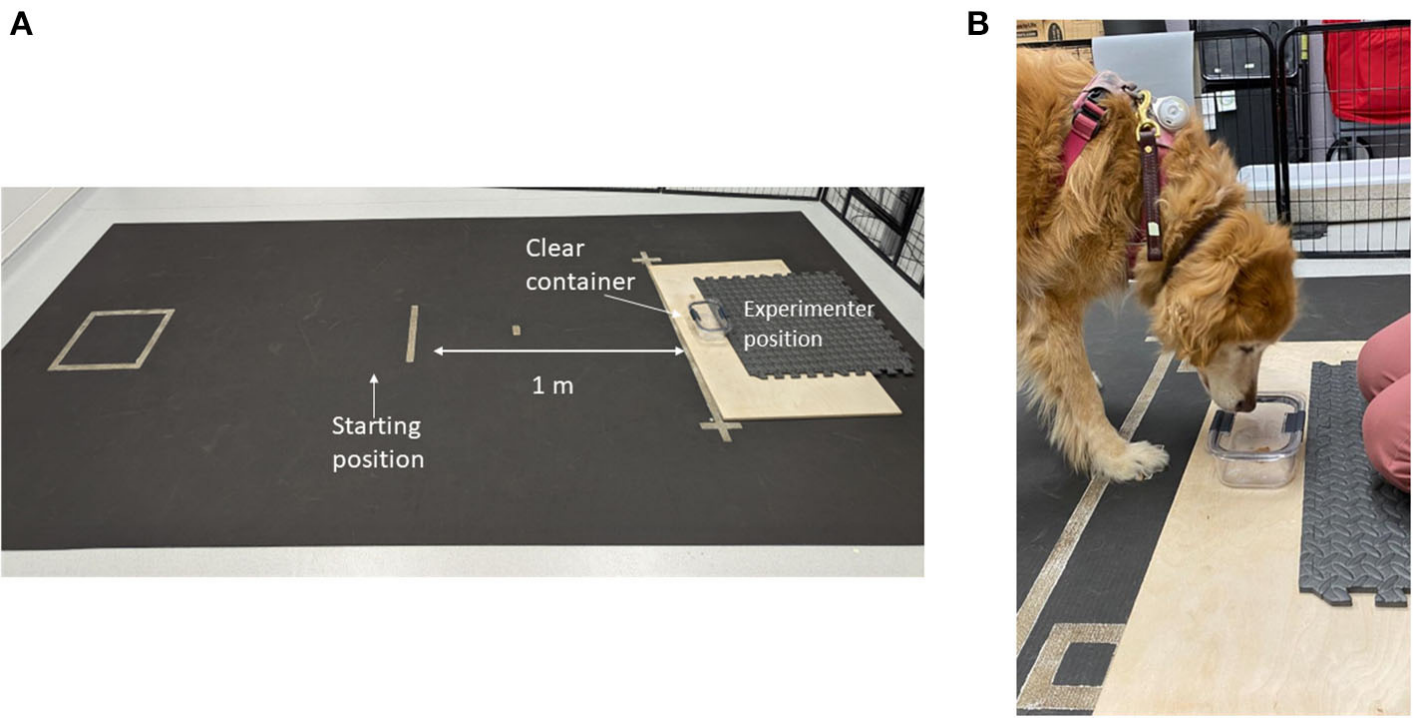
Photograph of the impossible task setup. **(A)** Depicts the testing field with starting location, container location and experimenter location. **(B)** Close-up photo of the subject interacting with the container.

Prior to testing, the subject was given a sample of the food reward (1” piece of chicken jerky) to ensure palatability. If the treat was refused additional options (Pupperoni^®^, kibble, various hard and soft treats) would be presented until the dog selected a highly palatable item. Each trial started with the dog on leash with a handler in a defined square 1 m from a clear glass container. The container was glued to a large wooden board which the experimenter kneeled on to prevent displacement during testing (Figure 1).

The handler held the dog in the starting square while the experimenter displayed a food reward to the subject before placing the reward in the open container. The top of the container was placed resting against the container for this trial. The experimenter then announced “Okay.” At this signal, the handler dropped the leash and turned around (back toward the experimenter) allowing the dog to obtain the treat reward from the container. Subsequent trials were performed with the clear lid placed on the container at 50 and 75% coverage over the container. During each of these trials, the dog was able to displace the lid off the container to obtain the treat. The testing trial could be performed once the dog obtained the treat with the lid at 75% coverage.

On the fourth (test) trial, the lid was clasped onto the container on all sides thus preventing access by the dog. The experimenter held a count-up stopwatch behind their back and followed the dog with their gaze to allow the dog to make eye contact at any point during the trial; the experimenter used the stopwatch to quantify the duration of eye contact. The handler held a count-down stopwatch set for 90 s for each trial. During the trial, the dog was allowed to freely move about the room and interact with the container or experimenter in any fashion. At the end of the 90 s, the trial was stopped, and the dog was given the treat from the container to ensure continued motivation for the reward. The testing period was recorded using overhead cameras to visualize the testing field on Amcrest video monitoring software. A video example of the impossible task can be found in the Supplementary material.

### Additional cognitive tests

Additional cognitive tests were performed as previously described (13). Cognitive tests included sustained gaze (measured as the number of seconds the dog would maintain gaze with the experimenter holding a treat near their face), cylinder tasks (inhibitory control and detour; measured as the percent of correct trials where the dog retrieved a treat from a transparent cylinder without touching the cylinder), working memory (measured as upper threshold, in seconds, that dogs could remember and select where a food reward had been hidden in a two-choice task), and social cues (pointing) tasks (measured as the percent of correct trials when the dog retrieved a food reward after the experimenter pointed to where it was hidden during a two-choice task). Full descriptions of these tests can be found in the Supplementary material. All cognitive tests were performed on the same day and included the impossible task, social cues, memory, sustained gaze, and cylinder tests. All tests were voluntary and not performed if the dog elected not to participate in the test (i.e., if the dog would not approach a task or leave the start box). If dogs appeared tired or disinterested during testing sessions, 10–30-min breaks were taken to maintain motivation and stamina.

### Video scoring

Trials were scored using Behavioral Observation Research Interactive Software (BORIS; Torino, Italy). Each trial was scored independently for time interacting with the container (seconds), time interacting with the experimenter (seconds), latency to disengage with the task (seconds), total time interacting with the task (seconds), and frequency of re-engagement with the task.

Time interacting with the container was defined as the time in seconds where the dog had either head or shoulders oriented toward the container while the dog was over halfway from the starting box to the container, physically touching the container, or sniffing directly around the container.

Time interacting with the experimenter was defined as the duration in seconds when the dog made eye contact with the experimenter from the half-way point between the starting box and the container and forward, or physically contacting the experimenter.

Latency to disengage from the task was defined as the first time in seconds when the dog did not engage with either the experimenter or the container for at least 3 s.

Total time interacting with the task was defined as the summation of time in seconds interacting with the container and interacting with the observer.

Re-engagement was defined as any sequence of behaviors after the first disengagement where the dog engaged the impossible task again for any length of time. This was measured in frequency of events.

All dogs were scored by the same individual. Nine randomly selected dogs were scored by a secondary scorer to assess for inter-rater reliability.

### Statistical analysis

Statistical analyses were done using JMP 16.0 (Cary, NC). Inter-rater reliability was assessed by calculation of intraclass correlation for total time interacting using measurement systems analysis (EMP method). A Shapiro-Wilk goodness of fit test was done to assess normalcy of each variable. Comparisons between demographics, survey results, cognitive test outcomes, and measures of engagement with the impossible task were made using multivariate analysis and a non-parametric Spearman's ρ test. Comparison between interactions with the observer and with the container were done using a Wilcoxon signed ranked test. As this was exploratory, no corrections were made for multiple comparisons; significance was set at *p* < 0.05.

## Results

### Inter-rater reliability

Intraclass correlation was high for total time interacting with the task at 0.98 out of a maximum value of 1. The mean difference between coders was 1.54 s with a maximum of 6.85 s and a minimum of 0 s.

### Demographics

Thirty-five dogs who were enrolled in a longitudinal neuro-aging study participated in the impossible task. Of these dogs, three were excluded due to the trial ending prematurely due to a timing error. Of the thirty-two dogs included, thirty-one were able to perform the working memory task and twenty-eight were able to perform the pointing task. All thirty-two dogs were able to perform the remainder of the cognitive tests. The dogs ranged from 10.1 to 15.6 years of age within 20% of their expected lifespan (Table 1).

**Table 1:** Summary data for all variables: median, range, mean, standard deviation, and population for all independent variables measured in this study.

**Item**	**Median** ** (range)**	**Mean** ** (SD)**	**N**
Age (years)	12.9 (10.1–15.6)	13.0 (1.39)	32
Fraction life span ratio (years)	1.04 (0.85–1.20)	1.03 (0.1)	32
CADES (total score)	13 (0–74)	19.4 (18.1)	32
CCDR (total score)	36 (34–48)	37.9 (4.4)	32
CFQ (total score)	0.47 (0.31–0.66)	0.47 (0.11)	21
Pointing cue (% correct)	91.7 (58.3–100)	88.1 (12.3)	28
Working memory (seconds)	40 (0–120)	44.5 (41.7)	31
Inhibitory control (% correct)	87.5 (12.5–100)	79.3 (26.1)	32
Spatial detour (% correct)	50 (0–100)	49.6 (27.3)	32
Sustained gaze (seconds)	24.8 (1.53–60.0)	26.3 (17.2)	32
Total interaction time (seconds)	70.1 (21.8–90.0)	64.5 (21.1)	32
Latency to disengage (seconds)	42.7 (9.0–90.0)	48.2 (29.1)	32
Interactions with container (seconds)	46.1 (13.0–90.0)	47.8 (21.5)	32
Interactions with experimenter (seconds)	11.5 (0–64.6)	16.9 (16.0)	32
Re-engagement (seconds)	2 (0–6)	1.9 (1.9)	32

19 breeds were represented in the population: American Staffordshire terrier (2), Australian cattle dog (1), Australian Shepherd (1), Basset Hound (1), Beagle (2), Border collie (2), Brittany spaniel (1), Cockapoo (1), Dachshund (1), German Shorthair Pointer (1), Golden Retriever (2), Great Dane (1), Irish Setter (1), Jack Russell Terrier (1), Labrador Retriever (3), Pomeranian (1), Siberian Husky (1); the remaining 9 dogs were mixed breeds.

### Interacting with the observer and interacting with the container

In previous studies, the impossible task outcomes have been the time interacting with the observer and the time interacting with the container, with these two measures often compared to each other (30, 31). For the current study, our interest was in total engagement with the impossible task reported here; results for time interacting with the observer and container individually are available in Supplementary material 2.

### Age

**Figure 2:**
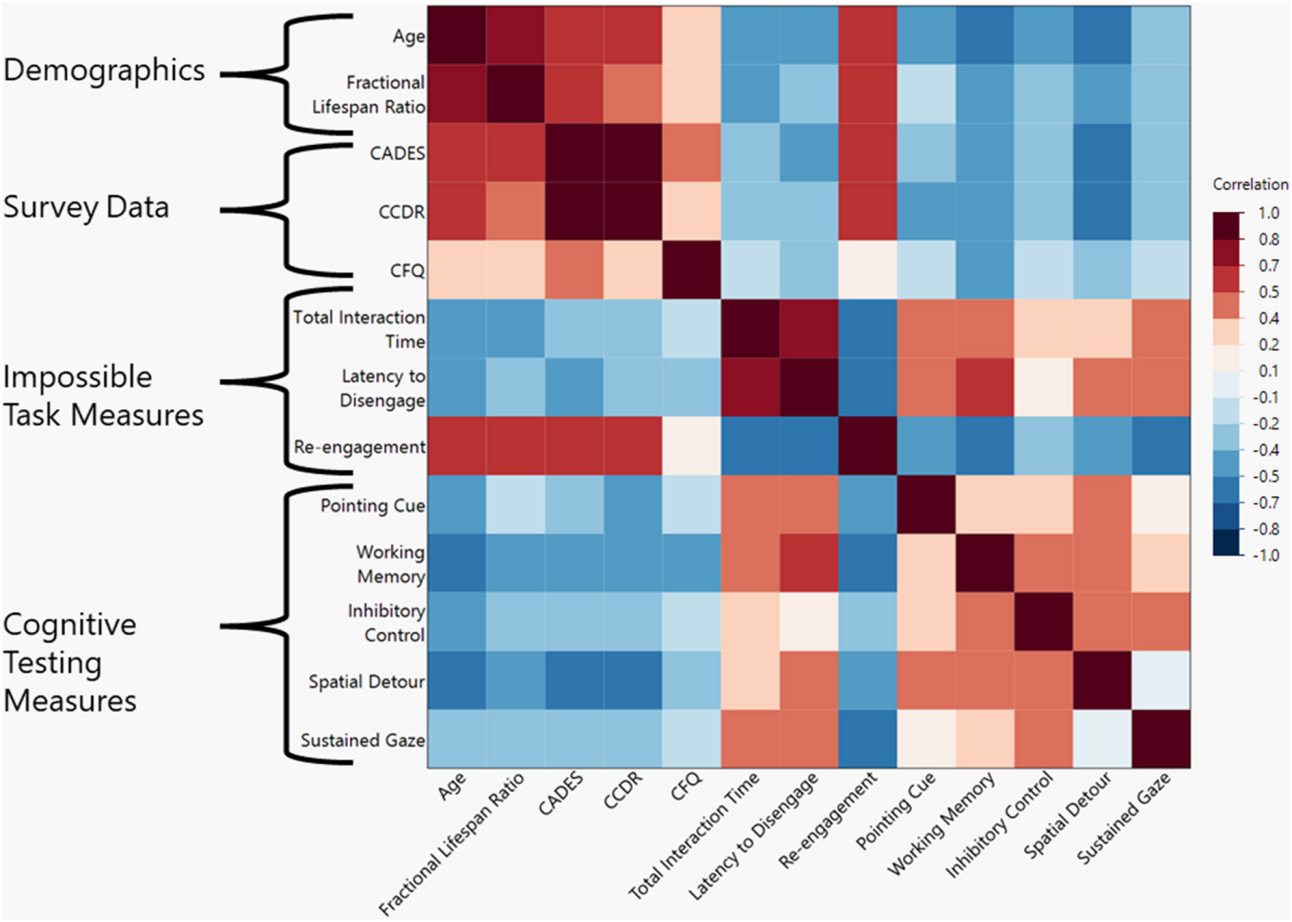
Heat map of correlations between demographic, survey data, impossible task measures, and cognitive test measures. Darker shades represent a higher Spearman's ρ-value with red shades representing positive correlations and blue shades representing negative correlations.

There was a negative correlation between the total age of the dog and the total time interacting with the task (ρ = −0.442, *p* = 0.011). A similar correlation was found when using fractional lifespan rather than age (ρ = −0.434, *p* = 0.015). For a visual representation of all correlations assessed in this study please refer to Figure 2.

There was a negative correlation between the latency to disengage from the task and the dog's total age (ρ = −0.404, *p* = 0.022) but not with the fractional life span ratio (ρ = −0.341, *p* = 0.061).

### CADES and CCDR

Dogs enrolled in the study tended to have mild signs of cognitive dysfunction as reported by owners. With the CADES survey, 7 dogs were considered normal, 14 were considered mildly impaired, 7 were moderately impaired, and 3 were considered severe. With the CCDR survey, 22 dogs were considered normal and 10 were considered at risk with no dogs scoring at the criterion for CCDS (Table 1). Total CCDR and CADES total showed a strong positive correlation (ρ = 0.877, *p* ≤ 0.001).

Total time interacting with the task had a negative correlation with the total CADES score (ρ = −0.35, *p* = 0.0496) indicating that as cognitive impairment increased, time interacting decreased. The total time interacting was not correlated with a specific subsection of the CADES questionnaire. Latency to disengage had a stronger correlation (ρ = −0.409, *p* = 0.0129) with the total CADES score and was positively correlated with the spatial subsection of the CADES questionnaire (ρ = −0.456, *p* = 0.009). When comparing the CCDR to the engagement with the impossible task, total time interacting showed a similar trend but was not significant (ρ = −0.267, *p* = 0.140), but latency to disengage was negatively correlated (ρ = −0.41, *p* = 0.040) with CCDR score.

### Canine frustration questionnaire

The total canine frustration questionnaire score (Table 1) did not correlate with any behavioral outcome, demographic, or questionnaire measure. The frustration coping section of the canine frustration questionnaire did show a positive correlation with the sleep subsection (ρ = 0.460, *p* = 0.036) of the CADES questionnaire as well as the total CADES score (ρ = 0.437, *p* = 0.048).

### Cognitive tests

Both total time interacting and latency to disengage (Table 1) were correlated with most cognitive tests performed except for the cylinder inhibitory control task, which was not correlated to either total time interacting (ρ = 0.278, *p* = 0.123) or latency to disengage (ρ = 0.202, *p* = 0.267). Overall, latency to disengage showed a stronger correlation when compared directly to the total time interacting with the task (Table 2).

**Table 2:** Correlations of total time interacting and latency to disengage with the impossible task with cognitive testing outcomes.

**Variable**	**By variable**	**Spearman ρ**	**Prob > |ρ|**
Total time interacting (seconds)	Pointing cue (% correct)	**0.401**	**0.030**
	Working memory (upper threshold seconds)	**0.530**	**0.002**
	Inhibitory control (% correct)	0.273	0.123
	Spatial detour (% correct)	**0.385**	**0.030**
	Sustained gaze (seconds)	**0.392**	**0.026**
Latency to disengage (seconds)	Pointing cue (% correct)	**0.479**	**0.010**
	Working memory upper threshold (seconds)	**0.672**	**<0.0001**
	Inhibitory control (% correct)	0.202	0.267
	Spatial detour (% correct)	**0.406**	**0.021**
	Sustained gaze (seconds)	**400**	**0.023**
	Total time interacting (seconds)	**0.742**	**<0.0001**

Spearman's ρ-value and significance of a multivariate analysis of the total time interacting with the task, latency to disengage from the task, and behavioral outcome measures. Significant correlations are shown in bold text.

### Re-engagement

Re-engaging with the impossible task was highly correlated with test performance across most measures (Table 3). The only measures that were not significantly correlated included the house-soiling section of the CADES questionnaire and the canine frustration questionnaire.

**Table 3:** Correlations of re-engagement with the impossible task with cognitive testing outcomes.

**Variable**	**By variable**	**Spearman ρ**	**Prob > |ρ|**
Re-engagement (frequency)	**Age**	**0.583**	**<0.001**
	**Calculated lifespan ratio**	**0.563**	**0.001**
	**CADES spatial score**	**0.585**	**<0.001**
	**CADES social score**	**0.381**	**0.032**
	**CADES sleep score**	**0.359**	**0.044**
	CADES soiling score	0.268	0.139
	**Total CADES score**	**0.562**	**<0.001**
	**CCDR**	**0.615**	**<0.001**
	**Pointing cue (% correct)**	**−0.434**	**0.021**
	**Working memory upper threshold (seconds)**	**−0.640**	**<0.001**
	**Inhibitory control (% correct)**	**−0.366**	**0.040**
	**Spatial detour (% correct)**	**−0.439**	**0.012**
	**Sustained gaze (seconds)**	**−0.578**	**<0.0001**

Spearman's ρ-value and significance of a multivariate analysis of the total time interacting with the task, latency to disengage from the task, and behavioral outcome measures. Significant correlations are shown in bold text.

## Discussion

This study employed a novel use of the impossible task to measure effects of a frustrating cognitive challenge in a population of senior dogs. Our primary objective with this study was to test the hypothesis that dogs who performed worse during a cognitive test battery and scored higher on cognitive impairment with owner directed surveys would show less engagement with the impossible task.

Overall, engagement with the impossible task was correlated with many behavioral outcomes and questionnaire data. Several tests have been studied in aging dogs to assess cognitive function, but are often constrained to research settings due to the amount and time and training required (3, 34). Rapid assessments of cognitive function have been suggested using food searching patterns, object manipulation, response to cues, and interaction with owners (16, 32, 35, 36). These tests have varying degrees of correlation with other cognitive dysfunction measures and feasibility to be performed in a clinical or at home environment. As CCDS is a multifaceted condition that affects several behavioral and cognitive domains, it is unlikely that any single cognitive test can be a true predictor of the severity of the cognitive decline. The impossible task requires little setup or equipment and can be performed in a short amount of time (~5 min) making this a feasible test in multiple environments with potential for use in a clirnically expedient battery of tests. Longitudinal data are needed to determine whether the interaction pattern changes within a dog as they age, and if this can be replicated outside of a controlled testing facility.

Latency to disengage from the impossible task and overall interaction with the task have not been previously studied as measures in this task. Our data set showed a more consistent correlation with other cognitive tests and questionnaire data when measuring the latency to disengage compared to the total time interacting with the task. A possible explanation for the difference between the two measures could be explained through the re-engagement evaluation. This measure was highly correlated with several cognitive tests as well as survey outcomes. The high level of reengagement with the task in dogs who were more cognitively impaired could represent dogs with impaired memory who were thus unable to remember they could not open the container after walking away. This pattern of interaction could also represent a perseverative or abnormal repetitive behavior which has been recognized as one of the first signs of cognitive decline in people (37).

As reviewed in Mendes, 2021 (31) the impossible task has typically been used as a cognitive test of communication and problem-solving. Our questionnaire data support this with the correlation between gazing at the experimenter and the pointing cue results, even though both these tests did not correlate with the social changes section in the CADES survey. Each individual cognitive test may highlight a particular domain of executive function (38), but likely requires multiple cognitive processes such as vision, attention, and frustration tolerance to be performed correctly. With the correlations seen between several other cognitive tests, the impossible task seems to capture multiple domains or highlight a common function used across cognitive testing.

A possible explanation for the high correlations could relate to the dog's overall ability to cope with frustration and unexpected challenges that are encountered during cognitive testing. We did not find that the impossible task correlated with the Canine Frustration Questionnaire. However, this is similar to previous findings that, apart from the frequency of vocalization, did not show correlations between behavioral measures and overall frustration score (29). While our experimental setup was not designed to capture the full range of vocalizations needed for appropriate analysis, during the impossible task dogs rarely expressed vocalizations (barking or whining) as a subjective observation by the experimenter. This could be the result of the test design. In previous tests of frustration, the dogs were prevented from interacting with the desired goal by a physical barrier (leash, door, and baby gate) (23, 29) and had restrictions to their overall movement (restraint or barrier to the desired area). In the impossible task, the dogs are free to interact with the container and have previously been successful at achieving their desired outcome leaving methods other than vocalizations as potential ways to influence the environment. The test is also short and in an otherwise stress-free environment which perhaps limits the intensity of the negative frustrative emotions. Our results are further supported by previous work evaluating vocalizations during the impossible task, where only 21% of dogs were found to express any vocalization (39).

While unexplored, another explanation for the observed correlations between the impossible task and other cognitive tests could be a resemblance between the impossible task and traditional measures of behavioral despair such as the forced swim and tail suspension test in rodents (40). In these tests, the animals are placed in an inescapable aversive environment and the amount of time the animal spends moving (thought to represent the attempt to escape) is measured. Animals in a depressive state will spend less energy and effort trying to escape that will then reverse once given an antidepressant medication. In the impossible task, the animal is again put in an unsolvable situation, although the desired outcome is obtaining a positive reward rather than escape. While the goal may be obtaining a positive reward, the frustrative state of being unable to obtain the reward may induce a temporary aversive state. Despite this difference, the end behavioral phenotype appears similar with the animal disengaging from the problem. In humans previous research has shown an increased risk of cognitive decline in individuals experiencing a high degree of negative affective states (41). In this study, it is difficult to interpret if this state is representative of despair or defeat in dogs, however, this could be analyzed by measuring engagement with the impossible task in dogs before and after administration of an anti-depressant medication, or by inclusion of a test of affect such as the judgement bias task. If dogs do experience true negative affect during this test, the question remains; is the negative affect contributing to cognitive decline or is cognitive decline causing the negative affect.

Limitations of this study included the cross-sectional nature of its design. As such we are only capturing a snapshot of the dog's cognitive capability which may vary from day to day. Several factors, apart from the influence of the dog's cognitive dysfunction, may contribute to the overall testing performance, including the amount of sleep, ongoing pain, and motivation for the food reward as well as many others; however, at present no studies have analyzed these potential confounding factors. Further research is needed to observe how these cognitive tests change over time within a given dog as well as with the changes to their health status. In addition, this study had a high proportion of dogs who were mildly or moderately impaired (per CADES score); ongoing work is being done to evaluate the impossible task in dogs with higher CADES and CCDR scores.

## Conclusions

This study presents a novel use of the impossible task to create a potentially frustrating task to assess cognition in aging dogs. By assessing the overall engagement with the task, we observed high correlations with other cognitive tests and questionnaires of owner perception of cognitive function. While the underlying etiology of the correlation remains unknown, potential explanations could include a decrease in frustration tolerance, poor sustained attention and memory, or susceptibility to enter a defeated or depressive state. Further research is needed to observe the interactions with the task as individual dogs age and to assess the validity of the test in a clinic or at-home environment.

## Data availability statement

The raw data supporting the conclusions of this article will be made available by the authors, without undue reservation.

## Ethics statement

The animal study was reviewed and approved by NCSU Institutional Animal Care and Use Committee. Written informed consent was obtained from the owners for the participation of their animals in this study.

## Author contributions

MK, NO, and MG were responsible for the design, analysis, and primary writing of the manuscript for this study. AM, KR, BC, HW, and GF all participated in the data acquisition. All authors participated in editing and review of the manuscript.
