# Supplementary material for: Relationship between engagement with the impossible task, cognitive testing, and cognitive questionnaires in a population of aging dogs
Source: Front Vet Sci. 2023 Jan 4;9:1052193. doi: 10.3389/fvets.2022.1052193 (PMC9848444; doi:10.3389/fvets.2022.1052193)
Supplement: Supplementary file 1 [file Data_Sheet_1.docx]

**Supplemental File 1. Additional Methods**

Excerpt detailing methods used in cognitive testing as published in: Fefer, G et al., 2022. Use of Cognitive Testing, Questionnaires, and Plasma Biomarkers to Quantify Cognitive Impairment in an Aging Pet Dog Population. Journal of Alzheimer’s disease: JAD 87, 1367–1378.

*Warm-ups and social cues (determines whether dogs used a communicative cue to locate a hidden food treat)*

Prior to the start of testing with social cues, all dogs were required to pass a warm-up phase. In this phase, a piece of food was placed on the mat by the experimenter while the dog was held by the handler at the starting line. After placing the food, the experimenter would say “okay” and the dog would be released to approach and consume the treat. This was performed until the dog completed 4 trials. Next, one red Solo® cup was placed on the mat. The experimenter picked up the cup, placed a food treat underneath (in view of the dog) and then said “okay” and the dog was released by the handler. When the dog approached the cup and either touched it or broke the plane of the cup, the experimenter would lift the cup and the dog would receive the treat. Using alternating sides, this was performed until the dog completed 4 trials. In the final phase of warm-ups, two identical cups were placed down, and the experimenter lifted one cup and placed a food treat underneath (in view of the dog). The experimenter then said “okay” and the dog was released by the handler to approach the cups. If the dog chose the correct cup (by touching or breaking the plane of the cup) then the experimenter lifted the cup, and the dog was able to consume the treat. If the dog chose the incorrect cup, the experimenter lifted the cup to display that there was no treat, and the dog was returned to the start line for another trial. Using alternating sides, this phase was conducted until the dog correctly retrieved the treat on 4/5 consecutive trials up to a maximum of 20 trials. If the dog did not succeed within the maximum number of trials, a short play break was taken, and warm-ups repeated. Dogs who completed the warm-ups moved on to the social cues. Dogs who failed to complete the warm-ups did not complete the social cue tasks and moved on to other tasks. For all social cue tasks, a food treat was hidden under one of two cups, out of view of the dog. The experimenter indicated (by pointing with the finger) which cup contained the hidden treat and said “okay” at which point the dog was released by the handler and allowed to approach the cups. When the dog selected the correct (indicated) cup, the food reward was given; when incorrect, they were calmly returned to the start line. Ten trials each were performed for the pointing and marker tasks; data were expressed as percentage of correct choices. To control for the potential use of odor to find the treat, both cups had a treat taped to the inside (inaccessible to the dog). As a further measure, an odor control task was performed where the food treat was hidden as before, but no indication was provided by the experimenter prior to releasing the dog. Eight trials were performed for the odor control task.

*Working memory task (Determines whether dogs could remember where a food treat was hidden after an increasing delay)*

Similar to the social cues tasks, two cups were placed on the mat and a small food treat was hidden under one of the cups (in view of the dog). After placing the treat, the experimenter would stand and start a timer for an incrementally increasing delay (up to 120 s). After the delay, the experimenter said “okay” and dogs were allowed to choose one of the two cups. When the correct cup was chosen, dogs received the food reward. Six trials were performed at each time delay. All dogs participated in the 3- and 6 s delays; after the 6 s delay, dogs who chose the correct cup on at least 4/6 trials moved to the next delay. There were up to nine delays tested: 3, 6, 10, 20, 40, 60, 80, 100, and 120 s. The number correct at each delay and the longest delay with at least 4/6 correct was recorded and dogs were then assigned one of three grades: grade 1: < 20 s, grade 2: 20–60 s, grade 3: > 60 s. The grades were used in statistical analysis.

*Cylinder tasks (inhibitory control and detour) (Assesses executive function through inhibitory control and adaptation to altered spatial contingency)*

In this task, dogs are asked to retrieve a food treat from the inside of a horizontal cylinder (open on both ends, cylinder height: 25.4 cm, cylinder width: 27.9 cm, cylinder diameter: 25.4 cm). First, familiarization trials were performed to ensure that dogs consistently retrieved the treat from the cylinder. The dog’s name was called, and a small food treat was placed in the opaque cylinder. Dogs were allowed to retrieve the hidden food and this procedure was repeated until participants retrieved the food four out of five times. Once this threshold had been reached, the cover over the cylinder was removed (making it transparent) and food was placed in the cylinder as in the familiarization trials. Dogs were released to approach the cylinder and retrieve the food. The dependent measure was whether the dog retrieved the food without touching the exterior of the cylinder over eight trials; the side they approached was also recorded. After eight trials, the side most often used by the dog was blocked with a plexiglass circle once the food treat was placed inside; over eight trials, dogs were allowed to approach the cylinder but had to retrieve the treat from their non-preferred side (detour). Data were expressed as a percentage of correct choices.

*Sustained attention/eye gaze (Determines the length of time dogs would sustain their attention toward a human face)*

Dogs were invited to make eye contact with the experimenter who called their name and held a food treat up near their eyes. The duration of sustained eye contact was recorded for a maximum of 60 seconds per trial. This was repeated three times for each dog and an average duration used for analysis.
