# Supplementary material for: Relationship between engagement with the impossible task, cognitive testing, and cognitive questionnaires in a population of aging dogs
Source: Front Vet Sci. 2023 Jan 4;9:1052193. doi: 10.3389/fvets.2022.1052193 (PMC9848444; doi:10.3389/fvets.2022.1052193)
Supplement: Supplementary file 2 [file Data_Sheet_2.docx]

# Supplemental File 2. Results

# *Interacting with the Container and Interacting with the Observer*

# Dogs interacted with the container significantly longer than with the observer (mean 47.8 seconds and 16.91 seconds respectively (Table 1); S=-220.0, p= <0.001). Time interacting with the container was significantly associated with the total time interacting with the task (ρ= 0.651, p=0. <0.001) and the latency to disengage from the task (ρ= 0.662, p=0. <0.001). Higher time interacting with the container correlated with a higher working memory (ρ= 0.482, p=0. 0.006), more correct responses in the cylinder spatial detour task (ρ= 0.458, p=0. 0.008), and lower disturbance in the sleep subsection of the CADES questionnaire (ρ= -0.414, p=0.018).

# Time interacting with the observer was not found to be statistically significant with either total time interacting or latency to disengage. Time interacting with the observer only showed a significant correlation with high correct responses in the social pointing cue (ρ= 0.381 p=0.045).
